# Stroke-Specific Swimming Critical Speed Testing: Balancing Feasibility and Scientific Rigour

**DOI:** 10.5114/jhk/170882

**Published:** 2023-11-28

**Authors:** Ben E. Scott, Richard Burden, Jeanne Dekerle

**Affiliations:** 1 Fatigue and Exercise Laboratory, School of Sport and Health Sciences, University of Brighton, Eastbourne, UK.; 2 English Institute of Sport, Loughborough University, Loughborough, UK.; 3 British Swimming, Loughborough University, Loughborough, UK.; 4 School of Sport, Exercise and Health Sciences, National Centre for Sport and Exercise Medicine, Loughborough University, Loughborough, UK.; 5 School of Sport, Health and Applied Science, St Mary’s University, Twickenham, UK.

**Keywords:** aerobic endurance, testing, swimming, high-performance, reliability, acceptability

## Abstract

This study aimed to assess the reliability of a two-distance critical speed protocol in the specialist strokes of national-level swimmers and understand the practical feasibility of extending the protocol to increase its validity. Thirty-two national-level swimmers (butterfly n = 7; backstroke n = 8; breaststroke n = 7; front crawl n = 10) swum three 200-m and three 400-m performance trials over a three-week period. Critical speed and supra-critical speed distance capacity were computed from the linear modelling of the distance-time relationship. Swimmers were subsequently asked whether they felt they could or would want to complete an 800-m trial as part of a three-distance critical speed protocol to enhance validity. Both 200-m and 400-m performances (coefficient of variation of < 2%) and derived critical speed (typical error of ≤ 0.04 m·s^−1^; coefficient of variation of < 4%) were reliable for all strokes, while supra-critical speed distance capacity (typical error from 4 to 9 m; coefficient of variation from 13 to 45%) was not reliable. Response rates to the follow-up questions were 100%. Few butterfly swimmers said they felt they could complete an 800-m performance trial (39%), with more positive responses for breaststroke (71%), backstroke (100%), and front crawl swimmers (100%). Butterfly swimmers were significantly less likely to say they could or would want to complete an 800-m trial than backstroke and front crawl swimmers (p < 0.05). Including a third distance 800-m trial to increase critical speed validity would not be acceptable to butterfly swimmers, would be challenging to breaststroke swimmers, but would be acceptable to front crawl and backstroke swimmers.

## Introduction

“Classic” critical speed (CS) testing uses modelling of the linear distance-time or curvilinear speed-time relationships to calculate CS and supra-CS distance capacity (D’) (Jones et al., 2019). Physiologically, CS should represent the lower boundary of the severe exercise intensity domain, separating an exercise intensity at which physiological homeostasis can be maintained from an exercise intensity at which it cannot (Bielec et al., 2010; ,Dekerle et al., 2010; Jones et al., 2019), and D’ should represent the work that can be done above CS (Poole et al., 1988). From a performance perspective, CS has been shown to only been sustainable for ~14.3 to 39.4 min (Dekerle et al., 2010), therefore previous attempts to define CS as a speed that could be sustained for ‘long periods of time’ or ‘forever’ without exhaustion (Wakayoshi et al., 1992, 1993) are imprecise and misleading (Zacca et al., 2010). The CS model should be of interest to high-performance swimmers and their coaches for several distinct reasons; it can be used to set training individualised intensities, to monitor the effectiveness of specific training blocks on short- and long-duration exercise tolerance, to predict competitive performances of 2- to 30-min duration as well as to compare the relative strengths and weaknesses of individual swimmers, yet its application poses unique challenges (Dekerle et al., 2006a, 2006b;Demirkan et al., 2023). Research suggests that a scientifically valid two-parameter CS model requires 3–7 performance trial efforts lasting ~2 to ~15 minutes (Dornowski et al., 2019; Poole et al., 2016; Zacca et al., 2010), with ~5-minute difference in duration between the shortest and longest trials (Bishop et al., 1998)and sufficient recovery between trials to minimise residual fatigue. Such duration is recommended to allow for *ṾO*_2max_ to be reached within each trial (Hill et al., 2002), a criterion for the valid estimation of CS. The number of trials recommended provides degrees of freedom and less error being introduced from one ‘bad’ trial than would be the case if completing just two trials. To apply these recommendations in front crawl swimming (Wakayoshi et al., 1992),a range of trial distances from 200 m to 1,500 m has been recommended (Dekerle et al., 2006a). In practice, such criteria pose significant practical challenges, particularly in a high-performance sport environment, as completing additional, longer distance performance trials causes further fatigue, may be feared to interfere with training adaptations, and disrupt an athlete’s training schedule. Further practical considerations need to be made for CS testing in ‘form stroke’ swimming (i.e., butterfly, backstroke, or breaststroke), which poses unique challenges that may affect the reliability, ecological and internal validity, and practical feasibility of CS testing.

Unlike front crawl swimmers who compete in distances up to 1,500 m in the pool, form stroke specialist swimmers only race distances up to 200 m. Furthermore, the actual energetic cost of swimming form strokes, especially simultaneous strokes (i.e., butterfly and breaststroke), is significantly greater than front crawl swimming (Barbosa et al., 2006; Capelli et al., 1998; Gonjo et al., 2018), which could contribute to greater technical breakdown over prolonged efforts. Together these factors may mean that form stroke swimmers, particularly simultaneous stroke swimmers, are less capable and potentially less motivated to complete CS protocols that include longer performance trial efforts in their competitive stroke. To the authors’ knowledge, only two studies have investigated the use of CS protocols using multiple performance trials in a form stroke—a validation of breaststroke-specific CS estimated using 50-m, 300-m and 2,000-m performance trials (Takahashi et al., 2009) and an assessment of anaerobic critical velocity using ultra-short 10, 15, 20 and 25 m distance trials (Marinho et al., 2011). No studies have investigated the reliability of stroke-specific CS protocols or assessed the potential trade-off that may need to be made with regard to the feasibility and acceptability of a protocol (Bowen et al., 2009) in order to maximise its validity.

To make CS estimation more practically feasible for front-crawl swimmers some researchers and practitioners have used only two relatively short distance performance trials, commonly a 200-m and 400-m combination (Dekerle et al., 2002; Wakayoshi et al., 1993). Interestingly, the reliability of this shortened procedure is currently unknown; laboratory-based and field-based test-retest design studies using critical power modelling would indicate that CS may be estimated more reliably than D’ regardless of the protocol (Hopkins et al., 2001; Triska et al., 2017). With regard to validity, the 200-m and 400-m combination leads to higher CS estimates than if longer trials were inserted in the distance-time model (di Prampero et al., 2008; Martin and Whyte, 2000; Toubekis and Tokmakidis, 2013). This is in part because of the relatively short trial duration, but also because in swimming, the non-linear relationship between energy cost and swimming speed affects the linearity of the distance-time relationship (Capelli et al., 1998; di Prampero et al., 2008). Including trials of ≥ 800 m will likely produce a slower CS with more criterion and internal validity (Dekerle et al., 2002; Martin and Whyte, 2000; Toubekis and Tokmakidis, 2013) than just performing shorter trials, but this comes at the cost of practical feasibility.

Variability in front crawl swimmers’ pacing profiles is known to increase as performance distance increases (Skorski et al., 2013) and variability in performance appears to be exacerbated in form strokes (Skorski et al., 2014) with a greater coefficient of variation (CV) of split times in the pacing of 200-m butterfly, backstroke, and breaststroke than 200-m front crawl. Together these factors could contribute to lower reliability of pacing when swimming a form stroke, particularly over longer distance efforts (McGibbon et al., 2018), which could in turn, affect performance and consequently CS/D’ reliability. However, presently, the actual reliability of CS/D’ estimation from protocols performed in the field is unknown in any stroke.

The aim of this study was to assess the reliability of CS and D’ calculated using 200-m and 400-m performance trials, and the performance pacing profiles for national standard butterfly, backstroke, breaststroke, and front crawl swimmers in their primary stroke. The study also aimed to identify the acceptability of implementing even longer distance efforts in a CS protocol. We hypothesised that (1) absolute and relative reliability of CS would be good (CV ≤ 5%, ICC ≥ 0.75) in front crawl swimmers; (2) D’ would not be a reliable parameter in any stroke (CV ≥ 5%, ≤ ICC 0.75); (3) simultaneous stroke swimmers would be less likely than front crawl swimmers to feel that they could or would want to complete an 800-m trial.

## Methods

### Participants

Thirty-two national standard swimmers provided written informed consent to participate in this study approved by the University of Brighton Research Ethics Committee with experimental procedures conducted in accordance with the Declaration of Helsinki, except for prior registration in a database. Participants’ characteristics are presented in Table 1. FINA points were calculated using the methodology proposed by the Fédération Internationale de Natation (https://www.fina.org/swimming/points).

**Table 1:** Participants’ characteristics.

	Butterfly (n = 7)	Backstroke (n = 8)	Breaststroke (n = 7)	Front crawl (n = 10)
Age (years)	19 ± 2	18 ± 2	18 ± 2	17 ± 2
Sex (male/female)	4/3	6/2	4/3	6/4
Body height (cm)	174.5 ± 7.9	179.2 ± 9.3	174.4 ± 8.3	178.7 ± 7.9
Body mass (kg)	68.2 ± 8.2	71.3 ± 9.7	65.5 ± 5.0	66.8 ± 7.6
Urine osmolality (mOsm·l^−1^)	740 ± 244	642 ± 332	692 ± 278	662 ± 229
Short/middle/long distance specialism	5/2/0	3/5/0	3/4/0	5/4/1
Main event PB FINA point equivalent	749 ± 61	719 ± 107	784 ± 74	718 ± 73
Individual medley swimmers	1	2	1	0

### Design and Procedures

Over the course of three weeks, participants were required to perform three 200-m and three 400-m performance trials in randomised order, each separated by at least 48 hours. Performance trials of 200 m and 400 m were chosen as they both fell within the recommended ~2 to ~15-minute duration of trials for CS estimation whilst reducing the demand on participants that including a third even longer distance trial might introduce. Trial distances also matched those of Wakayoshi et al. (1993). Trials were completed in each swimmer’s specialist stroke; individual medley swimmers chose which stroke they swam. Participants performed all their performance trials at the same time of day in a 25-m pool following a standardised 1-km warm up. The warm up consisted of 400 m (50 front crawl / 50 backstroke) descending 100’s off six minutes, 4 x 100 m specialist stroke drill, kick and swim efforts off two minutes each and 8 x 25 m specialist stroke build and speed efforts off 40 s. Participants recorded their diet in the 24 hours prior to the first performance trial and were asked to replicate this for all subsequent trials. Participants provided a urine sample before each trial for the assessment of urine osmolality (Osmocheck; Vitech Scientific, Horsham, United Kingdom). No dehydration state was detected and there were no differences in participants’ urine osmolality across their three 200-m or 400-m performance trials (*p* > 0.05). No feedback was given during or immediately after the performance trials.

Prior to testing, lane ropes were fixed using 5-mm stainless steel lane rope clamps (WRST-05; S3i Group, Doncaster, United Kingdom) and calibrated using Class III Accuracy 50-m measuring tape (Surveyors Tape; Draper Tools, Chandler’s Ford, United Kingdom). Each swim was recorded on a video camera (HC-X1000; Panasonic, Osaka, Japan) with analysis of lap splits performed retrospectively using proprietary analysis software (Hudson, 2014). To reduce parallax error, the camera was positioned half-way up the length of the pool, as far from the pool and in as elevated a position as the facility allowed.

Having completed all performance trials, participants were asked to respond to two questions with either a “Yes” or a “No” answer: (1) “Could you have completed a full 800-m effort in your stroke?”; (2) “Would you want to swim a 200-m, 400-m and 800-m effort over three separate days in order to have a valid measure of your critical speed and D’?”. These questions were generated by the researchers before critical evaluation by external researchers and physiology practitioners. The questions were considered to achieve their intended purpose, indicating face validity.

### Statistical Analysis

The SPSS software package (version 24, SPSS, Chicago, IL) was used for statistical analysis with data presented as means ± SD unless otherwise stated. Outliers and normality of distribution were examined using boxplots and the Shapiro-Wilk test, respectively. Outliers were windsorized to the next highest value prior to further analysis. Violations of normality were kept in and are reported in the results section. A one-way repeated measures ANOVA was used to assess differences between the three trials. Sphericity was checked using the Mauchly’s test, when the assumption of sphericity was violated significance was examined using Greenhouse-Geisser correction. Bonferroni correction was performed for all post-hoc analysis where the assumption of sphericity was not violated.

A published spreadsheet was used to calculate typical error of measurement (TEM), a measure of absolute test-retest reliability (Hopkins, 2015). TEM was divided by the trial mean and multiplied by 100 to calculate the CV. Smallest detectable individual change (SDC_ind_) and smallest detectable group change (SDC_group_) values were calculated from mean TEM to 95% probability using equations 1 and 2, respectively (Vet et al., 2011). The smallest worthwhile change (SWC) was calculated by multiplyingbetween-subject SD values by 0.2.


$$\text{Equation 1: }SDC_{ind}=1.96\text{ x }\sqrt{2}\text{ x }TEM$$

$$\text{Equation 2}:\;SDC_{group}=\frac{(1.96\text{x}\sqrt{2\text{x}}TEM}{\sqrt{n}}\text{ [}n,\text{ sample size]}$$

Relative reliability was assessed through intra-class correlation coefficient (ICC) estimates and their 95% confidence intervals (CI) were calculated based on a single-rater, absolute agreement, two-way mixed effects model (ICC 2,1) (Koo and Li, 2016). ICC values < 0.50 were considered indicative of poor reliability, 0.50–0.74 moderate reliability, 0.75–0.89 good reliability and ≥ 0.90 excellent reliability (Koo and Li, 2016). A Fisher’s exact test was performed to assess responses to follow-up questions regarding the acceptability of an 800-m trial.

## Results

### Performance Trials

200-m and 400-m performance trial data are presented in Table 2; they were normally distributed and contained no outliers. There were no differences in total time over the three 200-m or 400-m performance trials for any of the four strokes tested (*p* > 0.05).

**Table 2:** Test-retest reliability of 200-m and 400-m performance across three performance trials.

	Time (s)			
	Butterfly	Backstroke	Breaststroke	Front crawl
**200-m Trials**				
Trial 1	138.14 ± 8.19	134.07 ± 8.28	153.20 ± 10.10	124.24 ± 6.12
Trial 2	139.20 ± 7.33	135.58 ± 7.22	155.95 ± 11.77	126.51 ± 7.33
Trial 3	139.25 ± 7.19	134.82 ± 7.61	154.93 ± 10.00	124.69 ± 6.16
TEM 1–2 (% CV)	1.18 (0.85)	2.51 (1.86)	1.53 (1.00)	2.14 (1.71)
TEM 2–3 (% CV)	0.78 (0.56)	2.77 (2.05)	1.85 (1.20)	2.48 (1.98)
Mean TEM (% CV)	1.00 (0.72)	2.64 (1.96)	1.70 (1.10)	2.32 (1.85)
SDC_ind_ (% mean)	2.77 (1.99)	7.33 (5.43)	4.72 (3.06)	6.42 (5.13)
SDC_group_(% mean)	1.05 (0.75)	2.59 (1.92)	1.78 (1.16)	2.03 (1.62)
SWC (% mean)	1.52 (1.09)	1.54 (1.15)	2.13 (1.38)	1.31 (1.05)
ICC (95% CI)	0.98 (0.91–1.00)	0.91 (0.74–0.98)	0.98 (0.91–1.00)	0.90 (0.71–0.97)
**400-m Trials**				
Trial 1	302.67 ± 18.96	284.30 ± 15.42	324.59 ± 17.86	265.44 ± 11.85
Trial 2	305.06 ± 20.79	284.70 ± 17.31	327.39 ± 17.97	264.66 ± 13.64
Trial 3	303.60 ± 19.71	285.16 ± 15.72	322.70 ± 20.07	263.31 ± 14.00
TEM 1–2 (% CV)	4.96 (1.63)	2.03 s (0.71)	4.95 s (1.52)	3.31 (1.25)
TEM 2–3 (% CV)	1.46 (0.48)	2.17 s (0.76)	4.98 s (1.53)	3.28 (1.24)
Mean TEM (% CV)	3.66 (1.20)	2.10 s (0.74)	4.97 s (1.53)	3.30 (1.25)
SDC_ind_ (% mean)	10.14 (3.34)	5.82 s (2.04)	13.77 s (4.24)	9.14 (3.46)
SDC_group_(% mean)	3.83 (1.26)	2.06 s (0.72)	5.20 s (1.60)	2.89 (1.09)
SWC (% mean)	3.97 (1.31)	3.23 s (1.14)	3.73 s (1.53)	2.64 (1.00)
ICC (95% CI)	0.96 (0.86–0.99)	0.99 (0.96–1.00)	0.93 (0.78–0.99)	0.95 (0.85–0.96)

**Figure 1:**
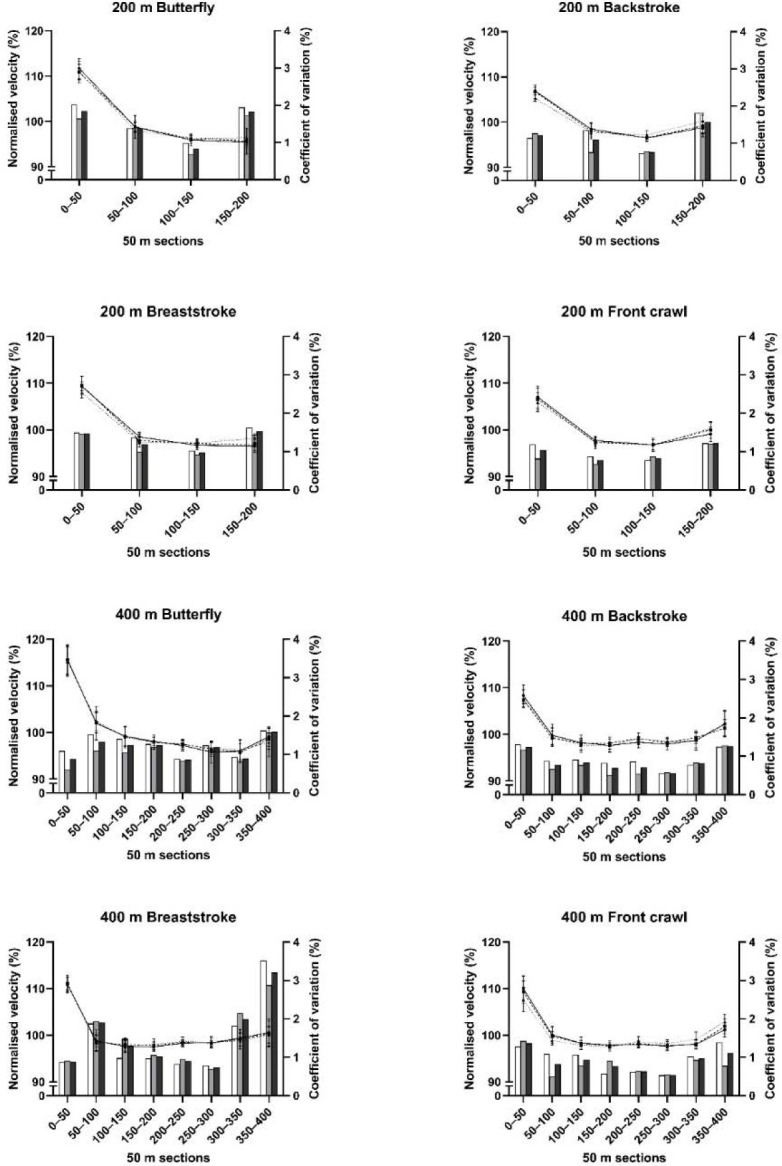
Pacing patterns data during performance trials. Performance trials 1 (solid line), 2 (dashed line) and 3 (dotted line represented as mean ± standard deviation, calculated from split times relative to mean velocity) and the coefficient of variation from trials 1–2 (white bars), 2–3 (grey bars) and overall (black bars) for all strokes and distances

Performance trial pacing data are presented in Figure 1. 50-m split CV were < 2.1% for all 200-m and 400-m trial combinations except the final 50 m of the 400-m breaststroke where the CV was 3.5% (trials 1–2), 2.9% (trials 2–3) and 3.2% (overall). The CVs for all 200-m and 400-m trials were higher in the final 50 m than the CV was at any point between 50 and 150 m of the 200-m trials or between 150 and 250 m of the 400-m trials.

### Critical Speed and Supra-CS Distance Capacity

CS and D’ data are presented in Tables 3 and 4, respectively. There were no significant differences in CS or D’ across trials for any of the strokes (*p* > 0.05). ICC analysis showed moderate to excellent relative reliability between CS calculated over the three sets of 200-m and 400-m performance trials (ICC ≥ 0.70). ICC analysis for D’ revealed poor relative reliability for backstroke, breaststroke and front crawl swimmers (ICC ≤ 0.70), but good relative reliability for butterfly swimmers (ICC = 0.76).

**Table 3:** Test-retest reliability of critical speed (m·s^−1^) across three performance trials.

	Critical Speed (m·s^−1^)			
	Butterfly	Backstroke	Breaststroke	Front crawl
Trial 1	1.22 ± 0.09	1.34 ± 0.07	1.17 ± 0.06	1.42 ± 0.06
Trial 2	1.21 ± 0.10	1.35 ± 0.12	1.16 ± 0.05	1.45 ± 0.08
Trial 3	1.22 ± 0.10	1.33 ± 0.08	1.20 ± 0.08	1.45 ± 0.09
TEM 1–2 (% CV)	0.03 (2.70)	0.03 (2.59)	0.04 (3.22)	0.04 (2.85)
TEM 2–3 (% CV)	0.01 (0.95)	0.04 (3.24)	0.04 (3.51)	0.05 (2.36)
Mean TEM (% CV)	0.02 (2.02)	0.04 (2.94)	0.04 (3.36)	0.04 (3.07)
SDC_ind_ (% mean)	0.07 (1.99)	0.11 (5.43)	0.11 (3.06)	0.12 (5.13)
SDC_group_ (% mean)	0.03 (0.75)	0.04 (1.92)	0.04 (1.16)	0.04 (1.62)
SWC (% mean)	0.02 (1.09)	0.02 (1.15)	0.01 (1.38)	0.02 (1.05)
ICC (95% CI)	0.90 (0.69–0.98)	0.88 (0.66–0.97)	0.70 (0.31–0.93)	0.71 (0.39–0.91)

**Table 4:** Test-retest reliability of D’ across three performance trials.

	D’ (m)			
	Butterfly	Backstroke	Breaststroke	Front crawl
Trial 1	31.69 ± 9.31	21.40 ± 7.63	15.19 ± 6.88	23.57 ± 4.58
Trial 2	31.55 ± 9.70	16.19 ± 14.94*	20.70 ± 9.11	17.85 ± 8.75
Trial 3	29.98 ± 9.92	21.30 ± 4.41	21.20 ± 7.67	18.90 ± 6.78
TEM 1–2 (% CV)	5.36 (16.95)	7.92 (42.13)	6.98 (38.92)	7.32 (35.35)
TEM 2–3 (% CV)	2.30 (7.47)	9.52 (50.81)	6.28 (29.99)	9.50 (51.69)
Mean TEM (% CV)	4.12 (13.27)	8.76 (44.62)	6.64 (34.91)	8.48 (42.17)
SDC_ind_ (% mean)	11.43 (36.77)	24.27 (123.67)	18.42 (96.76)	23.50 (116.90)
SDC_group_(% mean)	4.23 (13.90)	8.58 (43.73)	6.96 (36.57)	7.43 (36.97)
SWC (% mean)	1.93 (6.21)	2.00 (10.20)	1.59 (8.34)	1.38 (6.88)
ICC (95% CI)	0.76 (0.37–0.95)	0.40 (−0.22–0.81)	0.38 (−0.30–0.82)	−0.14 (−0.34–0.29)

* = D’ data calculated were not normally distributed

### Acceptability

**Figure 2:**
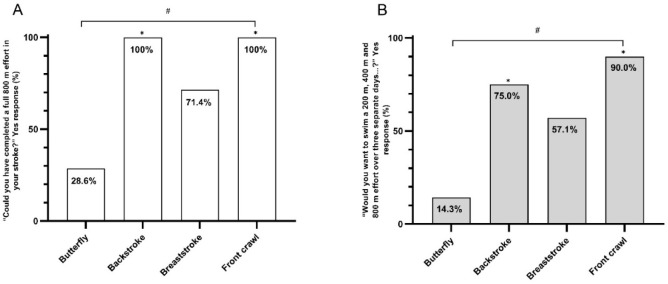
Percentage of participants who responded “yes” when asked whether they thought (A) they could have completed an 800-m trial, and (B) they would have wanted to complete an 800-m trial. * denotes difference from butterfly swimmers, # denotes overall difference across strokes (p < 0.05)

Proportions of swimmers who stated that they could (*p* = 0.001) or would want to (*p* = 0.013) complete an 800-m performance trial was influenced at the group level by stroke. Comparisons of individual stroke responses are presented in Figure 2. Butterfly swimmers were less likely than backstroke (*p* = 0.007) and front crawl swimmers (*p* = 0.003) to state that they could complete an 800-m trial. Butterfly swimmers were also less likely than backstroke (*p* = 0.041) and front crawl swimmers (*p* = 0.004) to state that they would want to complete an 800-m trial as part of a protocol to get a valid measure of their CS and D’. There were no statistically significant differences in the responses of breaststroke swimmers and those of swimmers of any other stroke (*p* > 0.05).

## Discussion

The main findings from this study were that both 200-m and 400-m performances (CV < 2%), and derived CS were reliable (TEM ≤ 0.04 m·s^−1^; CV < 4% for all strokes), while D’ was not reliable (TEM 4 to 9 m; CV 13 to 45%). Regarding protocol feasibility, few butterfly swimmers said they felt they could complete an 800-m performance trial (39%), with more positive responses for breaststroke (71%), backstroke (100%), and front crawl swimmers (100%). Butterfly swimmers were significantly less likely to say they could or would want to complete an 800-m trial than backstroke, and front crawl swimmers (*p*< 0.05).These findings are in agreement with hypotheses that absolute and relative reliability of CS would be good in front crawl swimmers, that D’ would not be a reliable parameter in any stroke and that simultaneous stroke swimmers would be less likely than front crawl swimmers to feel that they could or would want to complete an 800-m trial.

Potential applications of the CS model are wide-ranging (Dekerle et al., 2006a). Some of these applications, such as monitoring changes in an indicator of aerobic fitness, retain legitimacy even when a selected protocol overestimates “true” CS (i.e., the lower boundary of the severe intensity domain) but remains reliable, while other applications, such as setting accurate training zones, lose legitimacy. Likewise, as it has been reported, coaches only prescribe 22 to 36% of competitive form stroke swimmers’ training volumes in their specialist stroke, with most training being front crawl (Stewart and Hopkins, 2000). Some applications of the CS model remain useful and legitimate even when the protocol is not performed in a swimmer’s main competitive stroke, while other applications will lose their value. Practical application of the CS model in high performance swimming should therefore involve prior evaluation of a selected protocol’s validity, reliability, acceptability, as well as its intended application.

CS/D’ estimation in front crawl swimming has previously been assumed to be reliable because front crawl competition distance performance trials of 200 m to 800 m are reproducible (Pyne et al., 2004; Skorski et al., 2014). This may be a false assumption, as combining errors from multiple performance trials can result in a greater total error when calculating CS/D’. This is most likely when only two performance trials are used to make the protocol more practical, as one “bad” test will have a greater impact on the CS/D’ result than if averaging data from more trials (Dekerle et al., 2002; Wakayoshi et al., 1993). Assumptions of reliability instead of quantifying reliability in the form of SDC and SWC also limit the amount of practical information that can be gained when looking at assessing meaningful change.

To the authors’ knowledge, the reliability of ‘form stroke’ performance had only been examined up to competition distances of 200 m. The present study expands on this, demonstrating that performances over both 200 m and 400 m were highly reliable with the CV below 2% and the ICC ≥ 0.90 for all strokes. Mean TEMs were typically larger over 400-m (≤ 4.97 s) than 200-m performance trials (≤ 2.64 s), but CVs were similar. The absolute reliability of pacing in this study was good, with the CV of normalised velocity typically < 2% over each 50-m split, similar to Skorski et al. (2014). It is still unknown how reliable ‘form stroke’ performances over distances > 400 m would be, as may be advised if maximising the validity of CS estimation is prioritised over the practicality of completing longer trials.

It can therefore be expected that CS demonstrated very good, but weaker absolute and relative reliability (CV < 3.4%; ICC ≥ 0.70) than the 200-m and 400-m trials used for its calculation. Mean CVs obtained in this study were also larger across all strokes (~2 to 3%) than in studies examining reliability of CS estimation based on single or repeated effort all-out CS protocols (~1%) (Mitchell et al., 2018; Piatrikova et al., 2018). Using only two performance trials did not allow for the calculation of any error in CS estimation in this study—as a perfect linear distance-time relationship was the only possible outcome—and increased the potential effect of one ‘poor performance’. Despite this, CS calculated using 200-m and 400-m performance trials is still deemed sufficiently reliable to be used in practice for all swimming strokes in this study as the low CV and high ICC values evidence strong absolute and moderate to excellent relative reliability of CS in a test-retest scenario. The SWC values indicate that a CS change of 0.01 to 0.02 m·s^-1^ would be practically meaningful for performance in swimmers of all four strokes, while SDC_ind_ values indicate that 0.07 to 0.12 m·s^-1^ would be required to identify a ‘true’ change in an individual. This means swimmers from the sampled population could experience practically meaningful changes in their CS that would not be classified as a true change to a level of 95% confidence. The SWC values reported in this study will be more conservative than those relevant to an applied setting of national or international racing, because their calculation includes data from a mix of participant genders, race distance specialisations and relative abilities. For a more homogeneous sub-population, the resulting SWC would be narrower. It is therefore suggested that a sports scientist calculates the SWC that is most relevant to the individual swimmer and sample of swimmers (i.e., ‘squad’) they are working with where possible. Absolute and relative reliability of D’ (CV ~13–45%; ICC −0.14–0.76) is deemed not good enough to be of practical use in any stroke. Despite poor absolute reliability, the D’ of butterfly swimmers did show good relative reliability. This may well be a function of greater between-subject variability from butterfly swimmers in comparison with the other specialist stroke groups. Greater between-subject variability can have inflating effect on relative reliability, as within-subject variability is then comparatively low.

The deterioration of stroke length throughout longer duration performance trials at maximal efforts has been demonstrated, with the stroke rate increasing to compensate and help maintain speed (Laffite et al., 2004). This is likely particularly the case with breaststroke and butterfly swimmers where the energy cost of swimming is highest (Capelli et al., 1998). Using long duration efforts may therefore be particularly unrepresentative of race performance for sprint breaststroke and butterfly swimmers who may experience a significant breakdown in stroke mechanics. Although not within the scope of this paper, this is of interest and merits further investigation.

Including a third performance trial in the calculation of CS may reduce the level of random error, provide error estimates in CS and D’ calculations, and perhaps more importantly for the practitioner, enhance CS internal validity for an estimation of the lower boundary of the severe intensity domain (Jones et al., 2019). However, such a strategy would have to be traded off against the practicalities and acceptability of integrating this third testing session into a swimmer’s training plans. The present study shows that the inclusion of a maximal 800-m performance trial effort is unacceptable for butterfly swimmers and likely challenging for breaststroke swimmers. Instead, these swimmers could repeat the two-distance protocol or include shorter 300-m or 500-m performance trials as part of a three-distance protocol. Such an approach would introduce its own additional feasibility considerations and questions over calculated CS validity would remain since the longest trial duration would still be much shorter than the 15-min recommendation (Poole et al., 2016; Zacca et al., 2010). It is fundamental that swimmers are capable of or feel motivated enough to complete a longer repetition if this trial duration is to be introduced, as a lack of motivation may compromise the validity of the test. The data presented in this study show this not to be the case for butterfly and breaststroke swimmers with regard to an 800-m performance trial. It would be prudent for a coach to assess their individual swimmers’ willingness to undertake a third performance trial of any given distance to ensure buy-in and long-term compliance to such a testing protocol. Two butterfly swimmers for instance commented to the lead researcher before their first trial that they were unsure of being able to finish a 400-m effort, while one butterfly swimmer—a national record holder over 50 m—replied “DEFINITELY NOT” when asked whether they would swim an 800-m effort. Alternatively, rather than modelling the distance-time relationship of multiple performance trials, swimmers could complete a single or repeated effort all-out CS protocol such as the 12 x 25-m (Mitchell et al., 2018) or 3-min all-out (Piatrikova et al., 2018) tests. It is worth noting that the 12 x 25-m all-out test likely overestimates ‘true’ CS as it was validated against distance-time modelling of 100-m and 200-m race performances (Mitchell et al., 2018), while the 3-min all-out test has so far only been validated in front crawl swimming (Piatrikova et al., 2018). Despite their brevity, both tests also have their own feasibility considerations; the requirement of truly all-out swimming in these tests necessitates the highest levels of motivation and can make them both extremely stressful and unappealing testing options for some swimmers and their coaches.

In practice, CS modelled using the distance-timerelationship represents a practical and reliable method for assessing the aerobic capacity of national standard backstroke, breaststroke, and front crawl swimmers. The information provided in this study through TEM and SWC allows coaches and practitioners to make inferences related to the likelihood of swimmers’ having made practically meaningful changes in CS. Importantly, this protocol can be conducted with minimal need for specialist equipment or expertise making it highly practical in a swimming club setting. It is recommended that a coach or support staff hoping to use such a protocol first ensure they understand the theory and get the buy-in of swimmers they wish to use it with.

In conclusion the pacing of 200-m and 400-m performances demonstrates very good absolute reliability in national level swimmers, for all four strokes. A linear, two-parameter model using these two performance trials yields reliable CS, but not D’. Coaches and practitioners need to recognise the need for a balance between optimising the scientific robustness and acceptability of a CS protocol and how this may differ across strokes. The acceptability of including performance trials ≥ 800-m distance would be poor for butterfly swimmers and might also be challenging for breaststroke swimmers, as such they are not recommended for this purpose.
